# Superintelligence: A Crucial Juncture in the Development of Healthcare Sciences and Public Health

**DOI:** 10.3390/ijerph23050591

**Published:** 2026-04-30

**Authors:** Nicola Magnavita

**Affiliations:** Department of Safety and Bioethics, Section of Occupational Health, Università Cattolica del Sacro Cuore, 00168 Rome, Italy; nicolamagnavita@gmail.com; Tel.: +39-3473300367

## 1. The Development of Technology and the Needs of Humanity

Over the course of just a few decades, health sciences have developed and expanded at an ever-increasing pace. While the last two centuries saw the emergence, consolidation, and subsequent impact of antibiotic and vaccine therapy, psychiatric and psychological care, hormone therapy, biotechnology, information technology, and the beginning of genetic technologies, the first quarter of the 21st century has revealed the vast potential offered by artificial intelligence (AI) and telecommunications (TLCs) in, for example, the processing of datasets for highly efficient personalized medicine and its possible dissemination across all healthcare settings. Many effects of TLCs and AI in healthcare are already evident, and several contributions to this Special Issue (SI) predict that others will be observed soon. The aim of this SI is to share researchers’ perspectives on the future development of healthcare. In fact, the contributions exceeded our initial expectations and outline themes of great interest that have opened up vast areas for debate.

AI, i.e., the use of digital technology to create systems capable of performing tasks commonly thought to require human intelligence [1], has rapidly permeated many aspects of daily life. When we receive assistance from an online translator, we realize that conversational AI can speak human languages fluently and dynamically, demonstrating what appears to a true understanding of their many nuances. If we allow a navigator to help us choose the best route, it will do it by rapidly performing a personalized cost–benefit analysis based on machine learning algorithms for data analysis and decision-making. The massive datasets of texts and codes assembled over the internet in recent decades enable many commercial and open-source programs based on generative AI to produce human-like text, images, or music that depend on large language models (LLMs), i.e., systems based on deep learning (DL) and natural language processing technologies [2]. These processes fluently and accurately generate human language and seemingly understand the complexity of written sentences, engage in conversations, and generate creative outputs. Early models of self-driving cars with advanced capabilities for perceiving and processing complex sensory data and making real-time decisions in dynamic environments already exist, and self-driving devices will soon be available in medicine. DL modules based on an artificial neural network are already part of self-driving magnetic resonance (MRI) scanners, with autonomous AI controlling. In the future, the system will probably be provided with all contour drawings and preliminary measurements via fully automated inline picture analysis [3]. Data-driven automated laboratories, or self-driving laboratories based on machine learning and optimization algorithms under development, can significantly accelerate molecular and material discovery [4,5]. Similar innovations are being developed and applied in all fields of medicine. AI and related technologies are already capable of providing more accurate diagnoses, drug development in record time, and personalized treatments.

While technology has made these spectacular advances, humans have undergone changes that threaten our very survival. An uncontrolled rise in the population, estimated to reach a global peak of 9.73 billion (95% uncertainty interval of 8.84 to 10.9) by 2064 [6], and the corresponding exponential consumption of land and resources, particularly in areas such as Sahel and South-East Asia, have collectively contributed to global warming, first triggered by the early 20th century industrial revolution based on fossil fuels [7,8], and have ultimately paved the way for a food crisis and the intensification of migratory movements [9]. Despite this, the major world economies are searching for vital elements and new sources of fossil energy in areas such as the environmentally fragile Arctic and Antarctic [10], since policies to limit the use of fossil fuels and those to protect nature are often seen as excessively burdensome. Several countries around the world (e.g., South Korea, Thailand, the People’s Republic of China), and especially European nations, are experiencing a steep decline in birth rates [6,11]. Although a reduction in birth rates could contribute to improving economic performance in the short term by increasing the proportion of working-age individuals and thereby potentially increasing labor force participation and savings in addition to higher per-child investments and a better quality of life, it places the prosperity of future generations at risk [12]. In Western European countries, and others such as Japan, that have been experiencing declining birth rates since the second half of the 20th century, clear problems are emerging concerning the need to care for a growing number of elderly people, as well as individuals affected by chronic diseases. In the long term, we must expect a reduction in welfare resources due to a decline in the number of young people who comprise the workforce [13,14,15,16]. Moreover, this will occur at a time when numerous conflicts in many parts of the world have forced many governments to allocate resources to wars rather than to healthcare [17].

Notwithstanding this dramatic picture, technology appears to offer new and extraordinary opportunities for the improvement of public health. The use of modern technologies can offset the skyrocketing costs associated with chronic diseases in an aging population and the development of increasingly expensive drugs. Generative AI and integrated devices could enhance home care. In the workplace, new technologies could help prevent risks that are currently difficult to address, such as working in isolation, exposure to high temperatures, and working in confined spaces [18]. In the future, AI may make it possible for occupational doctors to create precision medicine in the workplace, for example, by identifying genetic defects that favor the onset of occupational diseases [19,20]. For the first time in human history, there are tools that could make living and working environments truly safe and healthy and improve quality of life for everyone.

## 2. New Technologies in Healthcare

This Special Issue examines the many interesting possibilities that new technologies have opened up in healthcare and public health. Considering that the rapidly increasing aging population has brought about a global rise in the prevalence of sarcopenia and musculoskeletal pain, two interconnected conditions that have an adverse effect on physical function, quality of life, and independence in older adults, Grosman and Kalichman [21] state that new technologies can significantly improve the picture if clinicians, policymakers, and researchers are ready to implement collaborative strategies, prioritize investments in integrated healthcare, and address significant knowledge gaps. Referring to the broader field of the entire population, Clark and Martins [22] observe that new statistical techniques applied to the large amounts of data made available from the latest “smart” devices and utilities may allow us to skillfully manage interrelations between sleep, sedentary behavior, and physical activity. Unlike conventional approaches that encounter difficulties due to perfect multicollinearity, these techniques can lead to advances in epidemiology with significant health outcomes for obesity, cardiometabolic health, cognitive function, physical fitness, quality of life, glycomics, clinical psychometrics, and mental wellbeing. It is easy to imagine the impact that these developments will have on public health and health promotion.

Furthermore, by integrating data from various potential sources (ranging from the aforementioned smart devices to more conventional datasets), AI can contribute to early detection and disease diagnosis, better decision-making, fewer medication errors, and enhanced prospects for clinicians. The application of AI-based clinical decision systems has the potential to improve patient outcomes through better diagnostic accuracy, optimized treatment selection, and a reduction in medical errors [23]. For instance, the extensive application of wireless and integrated technologies has shown real-life effectiveness in optimizing supportive care in pediatric intensive care units [24,25], thus improving the quality of life for parents and the effectiveness of care from professional providers [26]. Since the pandemic, the use of digital technologies, including mobile or nonmobile technologies or virtual reality, have proved effective in certain settings by guaranteeing regular follow-up for patients affected by neurodegenerative disorders [27], cystic fibrosis [28], and even diabetes [29]. In fact, these technologies can significantly enhance health promotion and disease prevention in an aging population, as well as in the general population. For this goal to become a reality, data collection and transmission technologies must be standardized and patient access to technological devices must be guaranteed. It is also essential that diagnosis be followed by treatment, which must also be accessible and constantly monitored. Achieving this integration is far from simple. In view of the current situation, the detection and treatment of chronic diseases in older adults represent a highly ambitious aim. For example, obstructive sleep apnea, a disease whose prevalence increases with age, affects 54% of the adult population in Italy, yet only 4% of cases are diagnosed and less than 2% are treated [30]. With regard to diabetes, while considering that data- and AI-driven systems have the potential to significantly transform diabetes care in the coming years and can be integrated into routine clinical practice, achieving the many possible benefits means addressing barriers to scalable adoption, including issues related to data availability and exchange, health inequality, clinician hesitancy, and regulatory challenges [31]. Therefore, the real problem is not making it possible for some people to access systems that allow for continuous monitoring of their clinical conditions, because this has been possible for some time. The real challenge is to rationalize the healthcare system and ensure that it can be used by all who need it. In this field, artificial intelligence combined with the remote transmission of large amounts of data could make a great contribution to the rationalization of healthcare services and effectiveness. Public health could reach everyone who needed assistance.

The new possibilities that innovative technology offers could also improve quality of life for elderly people. In this field, population growth and the transformation of family models are leading to public health needs that are difficult to meet with traditional methods. Masala and Giorgi [32] observe that AI and assistive robotics have the potential to revolutionize care for older individuals by providing innovative, personalized solutions tailored to an aging demographic. Recent developments in machine learning, natural language processing, and computer vision have facilitated tailored treatment strategies and effective remote monitoring. Ongoing health monitoring and prompt interventions enhance patient outcomes and prolong home-based care. Furthermore, research is being conducted on AI-powered assistive robots featuring advanced motion control and adaptive response mechanisms to enhance physical and cognitive health. Indeed, companion robots, frequently integrated with emotional AI, have proved to be effective in alleviating loneliness and fostering connectedness. Healthcare is already moving in this direction by implementing AI and AI-powered assistive robots to enhance triage procedures, automate administrative tasks, and support clinical decisions, and further developments are expected. While the People’s Republic of China and the United States are spearheading these revolutionary changes, biomedicine and healthcare require similarly innovative regulations. While most regulative interventions have been focused on the protection of personal data derived and generated during healthcare procedures, safe implementation of new technologies in healthcare settings requires the introduction of adaptable systems capable of assessing the use of AI in premarket development and of following a life cycle management strategy that includes frequent local post-market performance assessment. There needs to be a very transparent relationship between producers, sponsors, and regulators. Specific techniques are required to assess large language models and their applications. Furthermore, the demands of all health stakeholders, ranging from big businesses to start-ups, must be balanced. The generation and acquisition of health data (ranging from basic anthropometric data to the definition of a sort of ‘genetic passport’) could be used for a range of activities far exceeding basic healthcare procedures, including but not limited to health insurance or employers selecting “healthier” workers. Any framework for evaluating and regulating AI use to achieve financial optimization for developers, payers, and health systems must be centered on patient health outcomes and coordinated with international organizations, local governments, and other regulated industries [33].

Seixas and Chung [34] note that new technologies will be able to overcome the typical fragmentation of medical disciplines to create a new type of personalized, proactive, population-based, and human-centered medicine. The AI-WHOLE (Alignment, Integration, Workflow, Holism, Outcomes, Learning, and Equity) policy that will guide emerging technologies will truly place the individual at the center of medical action.

## 3. The Growth in Health Needs

The percentage of the population that could benefit from these advantages is still unknown. Inequalities in patient diagnosis, treatment, and billing have been found in evaluations of AI models stratified across subpopulations [35]. The creation and application of AI systems that are not sufficiently equitable can jeopardize the provision of nondiscriminatory healthcare. Chowdhury and Turin observe that health disparities among marginalized populations continue to exist in numerous developed nations [36]. Various systems of oppression converge to influence health outcomes. Social inequalities are biologically embodied, resulting in health discrimination. Chowdhury and Turin claim that researchers should not only simply point out disparities but also indicate significant, equity-focused solutions. Recent technologies could make a fundamental contribution to this objective. The rationalization of health spending criteria and resource distribution could be improved by using AI. Moreover, better management of the information network relating to health needs and interventions could highlight and help resolve current waste and inefficiencies. In healthcare practices, flawed business models and incorrect workflows can be corrected through the implementation of AI-driven tools. Healthcare organizations must reassess fundamental issues, including misaligned financial incentives (e.g., fee-for-service models), dysfunctional medical workflows (e.g., elevated patient readmission rates), inadequate care coordination among providers, fragmented electronic health record systems, and insufficient patient education and engagement strategies, particularly in conjunction with AI implementation. Healthcare needs to change its cultural outlook by perceiving AI as an enabler rather than a threat, by highlighting its potential to improve healthcare delivery and generate new employment opportunities, and by simultaneously underlining the importance of resolving fundamental operational issues. This essential transformation calls for the investment of financial resources but also underlines the significance of human capital, ongoing education, and an environment conducive to the integration of AI [37].

The technologies available today and their integration can bring about a rationalization of healthcare spending that is based not only on economic efficiency but also on a crucial respect for the human and solidarity-based purpose of healthcare services, representing another key opportunity. While the costs of implementing this AI integration are initially enormous, the expansion of the database and therefore the number of service users is an inextricable part of the development of these systems. Just as the first information revolution extended its networks to an ever-increasing number of people, AI has potential global benefits that cannot exclude lower-income countries.

Certainly, the current economic and political situation suggests that many of the possibilities offered by new technologies will not be readily accessible in low- and middle-income countries for some time. Nevertheless, this problem offers two opportunities. The first, which Mc Conkey [38] correctly points out, is the need to enhance the human capabilities of those dedicated to healthcare activities. The author observes that assisting individuals with disabilities, both children and adults, requires not only technical resources but also, and above all, human characteristics that are not always adequately appreciated. The leadership qualities that are needed in order to initiate and maintain community-based support are facilitated by mentoring and coaching, the empowerment of individuals, networking opportunities, and the enhancement of interpersonal and communication skills. These characteristics contrast sharply with prevailing practices in health and social care, where priority is given to professional expertise rather than real experience, to technical skills rather than compassion and empathy, and to person-centered “treatment” rather than the cultivation of community and personal self-reliance. In other words, the application of Taylorism criteria (typical of industrial work organization) to healthcare has definite limits. To achieve appreciable results in public health, it is crucial that new technologies adopt a humanistic approach.

The introduction of AI into healthcare has also influenced the traditional doctor–patient relationship. Maintaining this relationship is necessary in order to guarantee users’ acceptance and trust, so AI algorithms should provide high levels of explainability and inclusivity. To justify the use of these instruments, there should be strong evidence of their benefits to patients and healthcare services. When using machines in healthcare, patients’ autonomy and dignity must be maintained. To prevent accidents and harm from occurring, there should be discussion to establish responsible and accountable applications of AI in medicine. As technology develops, a new role for healthcare providers will emerge, and adjustments are unavoidable [39].

## 4. The Development of Superintelligence

As we all prepare to face the opportunities and problems posed by this evolution, technological development will continue to progress. What we now commonly call AI should be more correctly referred to as narrow AI. Research is now underway towards achieving a much more powerful and pervasive evolution: strong AI. Artificial general intelligence (AGI, or strong AI) is a next-generation AI system capable of cross-disciplinary learning and reasoning with the ability to make connections between different fields, understand the world, and learn and apply intelligence to solve problems as broadly and flexibly as a human would. This form of advanced artificial intelligence is already being implemented in many key areas. One of these critical areas is the availability of newer large-scale LLMs capable of processing natural languages and huge datasets that are much more efficient than the current ones. Multi-sensory AI will enable AGI to process and interpret multiple types of data (e.g., text, images, audio, and video) to perform tasks or make decisions. Advanced neural networks, consisting of deep learning software modeled on how neurons function within the human brain, but much more complex, powerful, and advanced than those currently available, are under study, and hardly a day goes by without some progress being made in this field. Neuromorphic computers, hardware systems inspired by the neural and synaptic structures of the human brain, are under construction. At the same time, the ways in which we calculate are also evolving. AGI is based on evolutionary computing, a form of algorithmic optimization inspired by biological evolution, capable of iteratively improving a population of candidate solutions by mimicking the process of natural selection for problem solving. There is an overall trend towards realizing AGI-generated programming, i.e., code, applications, and programming generated by AGI systems without human intervention. 

The major groups in this sector, as well as the superpowers, are actively engaged in the development of these technologies, which they consider an intermediate step towards the birth of Artificial Superintelligence (ASI). In addition to answering questions, ASI is an intelligence that has the potential to surpass humans in a wide range of domains, including physics, strategy, medicine, creativity, logic, emotional intelligence, and beyond. It will probably be able to instantly digest enormous quantities of data, reason across fields, learn from errors, become a better version of itself, create new scientific theories, produce perfect code, and perhaps even make moral or emotional decisions. ASI would be superior to humans in all cognitive tasks. ASI will be able to self-improve as it develops and learns and could become an inexhaustible, hyperintelligent superbeing: a near-perfect supercomputer available 24/7, capable of processing and analyzing any amount of data with a degree of speed and precision that we cannot yet comprehend. ASI systems will have cutting-edge cognitive functions and thinking skills more advanced than any human. ASI-generated inventions could lead to innovations such as new drugs, materials, and energy sources. Seamless integration would further enable intuitive interaction through natural spoken language or even mental commands, necessitating breakthroughs in human–computer interaction akin to technological singularity. 

Superintelligence does not yet exist. We are still in the early stages of what is called narrow artificial intelligence (AI) and are taking the first steps towards AGI. However, global superpowers and economic giants are vying for dominance over the ASI that will emerge from the integration of AI/AGI, telecommunications, cloud, and interoperability standards [40]. The race has begun, but it is not easy to determine how long it will take to achieve these goals, although some believe it might take only a year or two [41]. In the United States, concern that other superpowers might threaten its supremacy through military and energy applications of ASI has given rise to the so-called “Manhattan Project 2” modeled on the top-secret American-led research during the Second World War that led to the atomic bomb. Authoritative voices have spoken against this idea, fearing that the mere development of ASI could pose a grave threat to humanity [42].

In the military and defense spheres, ASI could be used to develop powerful, autonomous weapons, significantly increasing the destructive potential of war. Furthermore, malicious actors could exploit ASI’s advanced capabilities for harmful purposes, such as social control, data collection, and the perpetuation of prejudice. Ultimately, an ASI could also pursue objectives that are existentially harmful to humanity. While the actual use of ASI in real-world settings remains a plausible but not yet tangible threat, during 2026, the headlines of international newspapers did hint at it during the conflicts in Venezuela and Iran: algorithmic warfare, an innovative approach defined by Peter Layton in 2018 [43] as a combination of advanced computing, ‘big data’, analytics, AI, autonomy, and robotics to support, augment, or autonomously conduct military decision-making and operations across domains such as targeting, intelligence, logistics, and cyber operations.

We have reached a critical juncture where society must decide how to use new technologies. On the one hand, there is the possibility of a future of coordination and caution where nations and global agencies unite to avoid unforeseen risks and ensure that superintelligence is aligned with human interests. On the other hand, there is a scenario of acceleration and competition in which the race for technological supremacy could result in an uncontrolled spiral, with every country and every company ignoring regulatory and security risks in order to outdo the others. If ASI systems have only military applications, little benefit can be expected for public health. If new technologies are dominated by only a few individuals or states that use them to maximize their revenue, this will accentuate economic inequalities and their negative impact on public health. Development in this direction would lead to what Harari prophesied in 2011 [44]: on the one hand, an elite capable of prolonging their lives to the extreme but nevertheless fragile because of exposure to violence and accidental events, and on the other, a mass of individuals who feel like demigods but are increasingly angry and desperate.

Recent years have seen governments refrain from intervening in regulating developments in AI, cloud computing, and satellite networks. This inertia has led to the growth of private power centers that could compete with, absorb, and overtake institutional power centers. The latent conflict between collective and private interests could now emerge on account of factors such as the ecological crisis triggered by global warming, the intensive exploitation of resources, an aging population, and exoduses caused by wars. Any resulting superpower, whether it resembles the governments we know today or is a different form of technocracy, will have to address the issue of public health and will be able to do so with means far superior to those that we had at our disposal over the last century.

The contribution of new technologies to public health will largely be determined by the outcome of this underlying conflict. It is naive to think that if the distribution of healthcare products is entrusted to private companies, it will meet the needs of the population. The aim of companies is to make a profit, and if they pursue social wellbeing, it is mainly to improve their public image. Only in countries where healthcare is outsourced to private companies can large corporations have a vested interest in organizing and financing health promotion initiatives; however, they always pursue objectives that lead to short-term improvements in productivity or reductions in the healthcare costs borne by their companies. In countries where the healthcare system is state -funded (through taxes paid by companies), it is not worthwhile for companies to invest in promotion. Therefore, in some large companies, we can expect significant developments in AI- and TLC-based projects for the early identification of diseases that could cause corporate healthcare costs to soar. This development, while certainly desirable, may tend to neglect interventions whose beneficial effects will be attained after the end of working life. Furthermore, it is likely that only a few large companies will have the capacity to design cost-effective promotional campaigns that ensure their sustainability. Small companies will be excluded from this project due to objective difficulties in finding the economic and professional resources necessary to start the programs and monitor their results [45]. Moreover, the current legal framework for the application of AI in occupational settings may lead to an unwanted digital divide which could not only impair fair and equal economic development but also lead to a significant threat to workers’ health and safety. The European Union Artificial Intelligence Act (Regulation (EU) 2024/1689) has, in fact, defined limits for the potential application of smart devices in occupational settings, as well as the content and extent of individual health data that can be collected, shared, and eventually processed in order to identify and design rational and effective preventative options [46]. Therefore, in the absence of comprehensive planning, Total Worker Health © programs designed to integrate health promotion into occupational risk prevention, by virtue of the greater effectiveness of interventions that make use of new technologies, will probably end up accentuating the difference in health levels between those included in these projects and those excluded.

However, the immense potential for change associated with ASI goes beyond the issue of the fair distribution of resources and involves their use. The introduction of ASI into healthcare offers previously unheard-of possibilities for transforming population health management, treatment planning, and diagnostics, but it also poses serious risks if these systems are not appropriately matched with clinical goals and human values [47]. The risk of misuse and misalignment must be adequately foreseen [48]. Simply programming ASI according to human ethics and morality is a complex matter, as there is no universally accepted set of moral codes. This could lead to ethical dilemmas and potentially harmful consequences, especially if ASI begins to operate outside of human control. The immense capabilities of ASI could generate unpredictable and uncontrollable behavior. The ability of ASI to learn and adapt rapidly could make it difficult to predict its actions and prevent potential harm.

AI researchers, computer scientists, tech giants, and global governments must carefully consider the potential benefits and risks of ASI to ensure that this transformative technology is used responsibly and ethically for the betterment of humanity. The ethical problems linked to the development of new technologies are mostly problems arising from their misuse. Public health is a benefit that takes time to mature. Only a collective power shared by citizens can, through taxation, create funds to finance new technologies for the public good. New ways of distributing medical knowledge and health resources, new health promotion policies, and new methods of scientific research together with the application of these methods are available to humanity today. We will have to decide whether to make good use of them or whether they will become a tool for accelerating the end of homo sapiens. The superintelligence that is now being developed could enhance humanity’s ability to deal with crises. Clearly, controlling superintelligence and its orientation towards war or extra-profit purposes rather than towards health will be a crucial issue that we will have to face.

## Data Availability

No new data were created.

## Abbreviations

The following abbreviations are used in this manuscript:

AGI	Artificial general intelligence
AI	Artificial Intelligence
ASI	Artificial Superintelligence
LLMs	Large language models
MRI	Magnetic resonance
SI	Special Issue
TLCs	Telecommunications
